# Development and validation of an educational video for newly initiating peritoneal dialysis patients: from perioperative care to home-based management

**DOI:** 10.3389/fmed.2026.1654934

**Published:** 2026-04-10

**Authors:** Minfang Lv, Jing Wang, Xiaojuan Chen, Shuyan Li, Dan Li

**Affiliations:** Department of Nursing, The Second Affiliated Hospital, Zhejiang University School of Medicine, Hangzhou, China

**Keywords:** health education, patient, perioperative educational video, peritoneal dialysis, reliability and validity evaluation

## Abstract

**Background:**

Conventional education methods are insufficient to meet the increasing demand for patient health literacy. These methods require repeated explanations by healthcare personnel, and printed materials lack vividness, leading to poor patient comprehension. Consequently, these traditional approaches consume substantial healthcare resources and are inefficient.

**Aim:**

The objective of this study was to develop and validate an educational video tailored for the patients undergoing peritoneal dialysis (PD) catheter placement.

**Methods:**

This study adopted a methodological design. The research proceeded in five stages: (1) establishment of a multidisciplinary research team; (2) identification of key video topics and script development based on literature review, expert discussions, and patient interviews; (3) content reliability verification of the script via two rounds of Delphi consultation with 10 specialists; (4) production of the video; and (5) assessment of content validity by 20 newly initiating PD patients.

**Results:**

Following two rounds of expert consultation, the script content was validated. Subsequent patient validation confirmed the educational efficacy of the video. The final video covered 11 thematic areas: preoperative knowledge; fluid balance and dietary management; pharmacological knowledge; catheter care; exit site care; PD exchange procedures; home-based troubleshooting-related; home-based troubleshooting—daily life; home-based troubleshooting—infection; automated peritoneal dialysis (APD) operation; and rehabilitation guidance. The authority coefficient of all experts was ≥ 0.7, and Kendall’s coefficient of concordance exceeded 0.3 after both rounds (*p* < 0.01). Reliability and validity analyses demonstrated Cronbach’s *α* coefficients > 0.9 for all dimensions and overall content, with content validity index (CVI) values also > 0.9. Among the 20 feedback questionnaires collected from patients, 1 rated the video as qualified and 19 as excellent. Patients demonstrated satisfactory comprehension of both theoretical knowledge and practical procedures.

**Conclusion:**

This study developed and validated an educational video for the perioperative period of PD catheter placement. Through a multidisciplinary approach, expert Delphi consultation, and patient feedback, the video demonstrated high reliability and validity. Results showed it effectively enhanced patients’ understanding of relevant knowledge and procedures, offering a valuable tool to improve health literacy and optimize healthcare resource use.

## Introduction

Peritoneal dialysis (PD) is a commonly employed kidney replacement therapy for patients with chronic kidney failure (1). By instilling dialysate into the peritoneal cavity and utilizing the peritoneum as a semi-permeable membrane, PD facilitates the clearance of metabolic waste and excess fluid from the body. Compared to hemodialysis, PD offers the advantages of technical simplicity and reduced cardiovascular burden, and is thus widely adopted in clinical practice (2).

For patients newly undergoing PD catheter insertion, perioperative education is essential to ensure adequate understanding of dialysis exchange procedures, exit-site care, and home-based self-management. Catheter implantation is typically performed to establish a long-term and functional dialysis access route (3). However, due to a general lack of relevant knowledge and experience, many patients experience anxiety and uncertainty during the perioperative period, which may compromise surgical outcomes and elevate the risk of postoperative complications. Accordingly, comprehensive, and structured perioperative education is imperative.

In recent years, the application of video-based education—an intuitive and dynamic instructional medium—has expanded within healthcare setting (4). Video instruction enhances the delivery of complex information, improves patient comprehension and retention, and allows for repeated viewing, enabling patients to revisit key topics at their convenience. This supports improved understanding of perioperative precautions and strengthens self-care skills. Conventional education methods are insufficient to meet the increasing demand for patient health literacy in PD. While video-based education has been shown to improve knowledge retention and treatment adherence in various chronic diseases, its application in comprehensive PD management, particularly spanning from the perioperative period to home-based care, remains underexplored. Furthermore, with the growing integration of telehealth and digital resources into chronic disease management, developing accessible and validated video tools is essential to support patient self-management and optimize clinical outcomes.

The objective of this study was to develop a targeted perioperative educational video for newly initiating PD patients and to rigorously assess its reliability and validity. These two metrics are critical in evaluating the quality of educational materials: reliability refers to the consistency and stability of measurement outcomes, while validity pertains to the accuracy and relevance of the content. By assessing the video for reliability and validity, we aim to ensure that the material is both scientifically sound and practically effective in enhancing patient knowledge and self-management capacities during the perioperative phase, ultimately contributing to improved clinical outcomes and quality of life.

## Methods

**Figure 1:**
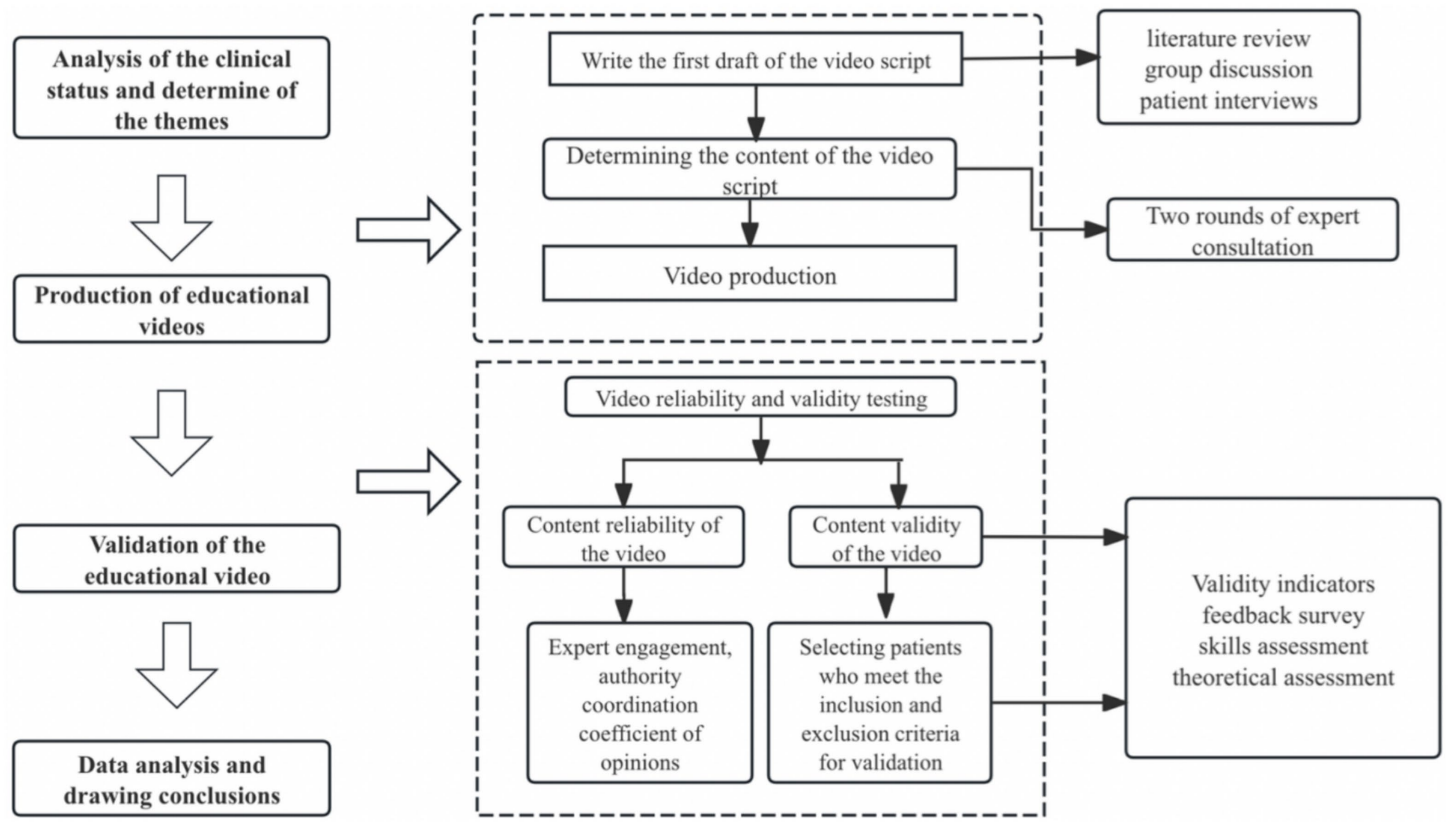
The study workflow.

This methodological study was conducted at the Second Affiliated Hospital of Zhejiang University School of Medicine from January 2024 to May 2025. The study was carried out in five stages: (1) assembling a multidisciplinary research team; (2) conducting literature retrieval, screening, and inclusion; (3) designing the content and drafting the script of the educational video; (4) confirming the script through expert consultation; and (5) validating the reliability and effectiveness of the educational video for newly initiating peritoneal dialysis patients. The study workflow see Figure 1.

### Step 1: formation of the research team

The research team was composed of five multidisciplinary experts. One nephrologist was responsible for defining the research topic; two specialists in nursing education and management were tasked with preparing the expert consultation questionnaires and selecting qualified panel members; and two graduate students oversaw organizing and analyzing the consultation responses. The two graduate students roles were primarily technical and coordinative, including: (a) assisting in literature searching and script compilation under the supervision of the multidisciplinary team; (b) coordinating meetings between experts and the production team; and (c) managing the logistics of video recording. All content decisions, including script finalization and educational messaging, were reviewed and approved by the multidisciplinary expert team. Each team member independently reviewed the content of each video module from their respective professional perspectives, offering suggestions for refinement to ensure scientific rigor and practical applicability. This study was approved by the Institutional Review Board of the hospital (Ethics Approval Number: (2025) Ethics Review No. 0639).

### Step 2: literature retrieval, screening, and inclusion

#### Literature search strategy

Following the hierarchical “6S” evidence pyramid model, a top-down search was conducted across multiple databases and websites, including Medlive Guideline Platform, UpToDate, Guidelines International Network (GIN), JBI Evidence-Based Healthcare Center, PubMed, CINAHL, Web of Science, Embase, Cochrane Library, Medline, CNKI, Wanfang Data, and the Chinese Biomedical Literature Database. Chinese search terms included “chronic kidney disease,” “peritoneal dialysis,” “PD patients,” and “peri-dialysis period,” as well as “health education” and “health promotion.” English search terms included “chronic kidney disease,” “peritoneal dialysis,” “health education,” and “education of health.” Both subject terms and free-text terms were used, and Boolean operators “AND” and “OR” were combined with wildcards to ensure a comprehensive search. The search covered the period from January 2013 to August 2024.

#### Literature screening

All retrieved references were imported into the NoteExpress reference management software for organization. Two graduate students with formal training in evidence-based medicine independently reviewed titles and abstracts to exclude obviously irrelevant studies. Full-text articles of potentially eligible references were further examined. In cases of disagreement, team members discussed to reach a consensus.

#### Inclusion and exclusion criteria

Inclusion criteria were: (1) study population consisted of patients with chronic kidney disease undergoing PD, aged >18 years; (2) the research content involved health education or promotion for PD patients; (3) study types included clinical decision-making tools, evidence-based guidelines, best practice summaries, expert consensus statements, systematic reviews, meta-analyses, evidence syntheses, and clinical trials; (4) studies were published in Chinese or English. Exclusion criteria were: (1) conference abstracts, case reports, letters, and editorials; (2) studies without full-text access; (3) guideline interpretations or research protocols only; (4) duplicate publications.

#### Results of literature inclusion

A total of 1,230 articles were initially retrieved, of which 1,220 were excluded based on the eligibility criteria. Ten articles were ultimately included: four clinical decision-making studies (5–8), one systematic review (9), and five clinical experimental studies (10–14). The screening process is illustrated in Appendix 1.

### Step 3: designing video content and drafting the initial script

Based on the included literature, the educational video content was designed, and an initial script was drafted. After discussions within the research team, eleven core video topics were finalized: preoperative knowledge; fluid balance and dietary management; pharmacological knowledge; catheter care; exit-site care; peritoneal dialysis exchange procedures; home-based troubleshooting—exchange-related; home-based troubleshooting—daily life; home-based troubleshooting—infection; automated peritoneal dialysis (APD) operation; and rehabilitation guidance. A draft script was developed under each thematic section (full content available in Appendix 2).

### Step 4: script validation via expert consultation

A Delphi questionnaire was developed based on the initial script for the perioperative educational video tailored to newly initiating PD patients. Expert inclusion criteria were: (1) clinical or nursing professionals with expertise in PD; (2) holding at least a bachelor’s degree and intermediate to senior professional titles; (3) having ≥8 years of experience in the PD field (In Chinese healthcare context, >8 years typically corresponds to professionals who have achieved at least an associate senior professional title or a doctoral degree with extensive clinical exposure); (4) high motivation and willingness to participate in multiple consultation rounds. Ten eligible experts from tertiary hospitals in Hangzhou, Zhejiang Province, were selected: three nephrologists (senior-level), three nursing administrators (senior-level), three PD-specialized nurses (supervisor level or higher), and one senior clinical head nurse in nephrology (≥15 years of experience). Responses were double-entered into Excel and screened according to predefined inclusion thresholds: mean score > 4.0, coefficient of variation < 0.25, and full-score proportion > 20% (15). After two consultation rounds, expert opinions converged, and the script was revised accordingly to generate the definitive version.

### Step 5: validation of the reliability and effectiveness of the perioperative educational video for newly initiating PD patients

The Analytic Hierarchy Process (AHP), a systems analysis method developed by Professor Saaty of the University of Pittsburgh in the 1970s (16), was employed to analyze the relationships among content items within the video script. Mean importance scores were used for pairwise comparisons among evaluation indicators. The scale was constructed using interval values, with the recommended 1–9 scale as proposed by Saaty.

Based on the finalized script, a professional animation designer was commissioned to create the visual characters, with the final imagery determined through iterative discussions among team members. Video production software was used to develop educational videos. A structured feedback questionnaire (Appendix 3) was also created to assess content validity. A five-point Likert scale was adopted, ranging from “not important at all” to “very important.” Internal consistency reliability was assessed using Cronbach’s *α* coefficient. Content validity was examined through both expert-rated content validity index (CVI) and structural validity analysis. In April and May 2025, twenty newly initiating PD inpatients from a tertiary hospital in Zhejiang Province were recruited for evaluation. Inclusion criteria were: (1) age ≥18 years; (2) first-time PD catheter placement due to acute or chronic kidney disease; (3) informed consent and voluntary participation; (4) primary school education or above. Exclusion criteria included: (1) presence of severe comorbidities; (2) speech, communication, or hearing impairments; (3) expected PD discontinuation within the next 6 months; (4) inability to operate a smartphone. The 20 participants ranged in age from 26 to 70 years: 5 were aged 26–30 years, 1 was 31–40 years, 3 were 41–50 years, 6 were 51–60 years, and 5 were over 60 years. There were 14 male (70%) and 6 female (30%) participants.

### Statistical analysis

All data were analyzed using SPSS version 27.0. Categorical variables were described as frequencies and percentages, and continuous variables were expressed as mean ± standard deviation (x̄ ± s). The reliability of expert consultation was assessed via measures of central tendency, authority coefficient, and Kendall’s coefficient of concordance. Reliability and validity tests were performed to evaluate the indicators. A two-sided *p*-value < 0.05 was considered statistically significant.

## Results

### Expert participation and authority

In both rounds of expert consultation, 10 questionnaires were distributed and fully recovered, yielding a 100.00% effective response rate. In the first and second rounds, 60 and 40% of the experts, respectively, provided specific feedback. The authority coefficients (Cr) for the two rounds were 0.808 and 0.814, respectively, indicating a prominent level of professional credibility among the participating experts.

### Coordination of expert opinions

Kendall’s coefficient of concordance for the two rounds of expert consultation was 0.167 and 0.315, respectively, both showing statistically significant differences (*p* < 0.001), which reflects increasing agreement among expert evaluations through iterative consultation.

### Finalization of video content for perioperative education in newly initiating PD patients

Based on two rounds of Delphi consultation, the final educational content of the perioperative video for newly initiating PD patients was confirmed. The video encompasses 11 overarching educational themes and 41 specific subtopics, with individual lengths ranging from 0 to 3 min. These 11 themed videos were sequentially played to patients 1 day before catheterization and on days 1–10 after catheterization. The playback sequence is shown in Table 1. The Analytic Hierarchy Process (AHP) was employed to calculate the individual and composite weights for each script item. All consistency ratios (CRs) of the content items were below 0.01, indicating the rationality of the assigned weights (see Table 1).

**Table 1:** Weight and composite weight of content items in the perioperative educational video for newly initiating peritoneal dialysis patients.

Item	Importance score (x̄ ± s)	Coefficient of variation	Composite weight
1. Preoperative knowledge	4.47 ± 0.52	0.12	0.009
1.1 Kidney physiology: removal of metabolic waste and excess fluid, maintenance of electrolyte balance, and blood pressure regulation	4.67 ± 0.49	0.1	0.0094
1.2 Definition of kidney failure: a syndrome caused by multiple etiologies leading to kidney dysfunction and metabolic disorders with multi-system involvement	4.87 ± 0.35	0.07	0.1085
1.3 End-stage kidney replacement therapies: kidney transplantation, peritoneal dialysis (PD), hemodialysis (HD)	4.80 ± 0.41	0.09	0.0189
1.4 Principle of PD: using the peritoneal membrane to eliminate waste products and fluid from the body	4.60 ± 0.63	0.14	0.0181
1.5 Types of PD: Continuous Ambulatory Peritoneal Dialysis (CAPD), Daytime Ambulatory Peritoneal Dialysis (DAPD), and Automated Peritoneal Dialysis (APD)	4.80 ± 0.41	0.09	0.0189
2. Peritoneal dialysis exchange procedures	4.87 ± 0.35	0.07	0.0192
2.1 Preparation before exchange: equipment preparation, personal readiness, and hand hygiene	4.13 ± 0.64	0.15	0.0163
2.2 Requirements for an environment exchange, item names, warming of dialysate, and proper fluid inspection	4.33 ± 0.72	0.17	0.0171
2.3 Dialysate connection techniques: preparation, connection, drainage, flushing, infusion, and disconnection	4.73 ± 0.46	0.1	0.0689
2.4 Post-exchange fluid evaluation: weighing, recording, and waste disposal	4.60 ± 0.51	0.11	0.0077
3. Exit-site care	4.60 ± 0.63	0.14	0.0077
3.1 Definition of exit site: location where the PD catheter exits the abdominal wall through the skin	4.60 ± 0.51	0.11	0.0077
3.2 Early phase (<6 weeks): secure catheter and ensure sterile dressing; late phase (>6 weeks): inspect and change dressings as required	4.53 ± 0.64	0.14	0.0076
3.3 Bathing guidance: no bathing within 2 weeks post-surgery; shower with protective cover at 2–6 weeks; unrestricted showering after healing at ≥6 weeks, but tub bathing remains prohibited	4.60 ± 0.51	0.11	0.0077
3.4 Dressing change procedure: wear mask and perform hand hygiene (performing the standardized seven-step handwashing technique first, and then using clean hands to retrieve a clean, sterilized surgical mask); prepare materials; remove dressing; Step 1—observe for redness/swelling; Step 2—palpate along tunnel; Step 3—press for exudate; rewash hands; disinfect in circular motion from inside out, exceeding dressing area; air-dry and reapply sterile cover and secure extension tube	4.53 ± 0.64	0.14	0.0076
3.5 Recognition of exit-site infection: localized redness, swelling, tenderness on palpation, and purulent discharge	4.33 ± 0.49	0.11	0.0073
4. Catheter care	4.67 ± 0.49	0.1	0.0078
4.1 Function of PD catheter: serves as a fixed channel for dialysate inflow and outflow; non-replaceable	4.67 ± 0.49	0.1	0.0078
4.2 Importance of understanding the catheter’s role	4.73 ± 0.80	0.17	0.0462
4.3 Protection strategies: perform hand hygiene before handling, always secure the catheter to the skin, avoid using scissors near the tubing	4.60 ± 0.63	0.14	0.0047
5. Fluid balance and dietary management	4.33 ± 0.49	0.14	0.0045
5.1 Importance of fluid balance: clinical manifestations and risks of fluid overload or dehydration	4.40 ± 0.74	0.14	0.0045
5.2 Fluid control strategies: monitor weight, measure blood pressure, restrict fluid intake, and use high-concentration dialysate as necessary	4.2 ± 0.78	0.11	0.0043
5.3 Recommended foods: high-quality animal proteins, vitamin-rich and fiber-containing foods	4.47 ± 0.64	0.1	0.0046
5.4 Restricted foods: high-phosphorus, high-potassium, and high-sodium foods (daily salt intake <3 g); sugary and fatty foods should be limited	4.60 ± 0.63	0.16	0.0047
5.5 Dialysis adequacy assessment: evaluation of treatment sufficiency	4.60 ± 0.51	0.14	0.0047
6. Medication knowledge	4.73 ± 0.46	0.09	0.0049
6.1 Common medications: antihypertensives, erythropoiesis-stimulating agents (ESAs), iron supplements, heparin, vitamin D, and others	4.27 ± 0.70	0.11	0.0044
6.2 Phosphate binders: to be chewed with meals (except sevelamer)	4.60 ± 0.63	0.17	0.0047
6.3 Function and storage of ESAs: corrects anemia; requires refrigeration	4.80 ± 0.41	0.11	0.0689
6.4 Antihypertensive administration principles: monitor blood pressure regularly; avoid arbitrary dosage adjustments	4.60 ± 0.51	0.09	0.0352
7. Simulated home-based dialysis problems—exchange abnormalities	4.40 ± 0.74	0.07	0.0337
7.1 Infusion or drainage difficulty: tubing kinking, air bubbles, fibrin formation, improper posture; ensure infusion bag is elevated above the abdomen	4.87 ± 0.52	0.07	0.1085
7.2 Management of constipation: use stool softeners, engage in physical activity, consume high-fiber foods	4.80 ± 041	0.09	0.0141
7.3 Abnormal dialysate appearance: red (related to menstruation or vigorous activity—observe if minimal, flush immediately if excessive); cloudy effluent requires immediate medical attention	4.87 ± 0.35	0.18	0.0143
7.4 Dry contamination of materials: replace dialysate bag if outlet is contaminated; replace connector or povidone-iodine cap if inner surface is contaminated	4.87 ± 0.35	0.14	0.0143
7.5 Dialysate leakage: (1) double-line tubing rupture—clamp damaged area, replace dialysate; (2) loose connector—clamp proximal end, replace connector; (3) catheter rupture—clamp proximal to break, return to center; (4) exit-site leakage—drain cavity, apply sterile gauze, seek prompt treatment	4.80 ± 0.41	0.2	0.0141
7.6 Dislodged titanium adapter or extension tube during exchange: immediately clamp proximal catheter with blue clip and return to dialysis center	4.53 ± 0.83	0.14	0.0133
8. Simulated home-based dialysis problems—infection	4.07 ± 0.80	0.05	0.0135
8.1 Signs of peritonitis: cloudy effluent, abdominal pain, fever	4.47 ± 0.64	0.11	0.0119
8.2 If peritonitis is suspected: seek immediate medical attention and bring a full drainage bag for testing	4.93 ± 0.26	0.12	0.0077
8.3 Exit-site infection: contact dialysis center immediately; report any pain along the subcutaneous tunnel	4.60 ± 0.51	0.2	0.0076
9. Simulated home-based dialysis problems—daily care	4.13 ± 0.52	0.12	0.0073
9.1 Constipation: may hinder dialysate flow and increase risk of infection; manage with fiber intake, exercise, and medication	4.13 ± 0.83	0.14	0.0078
9.2 Skin itching: avoid high-phosphorus foods, use phosphate binders, refrain from scented soaps or strong cleansers; apply moisturizer after showering	4.40 ± 0.51	0.21	0.0078
9.3 Hernia: may require surgical repair; contact dialysis center if present	4.40 ± 0.63	0.1	0.0462
9.4 Identification of anemia and bone disease symptoms	4.20 ± 0.86	0.18	0.0047
10. Automated peritoneal dialysis (APD) operation	4.73 ± 0.46	0.11	0.0045
10.1 Equipment preparation: necessary items for home-based machine-assisted dialysis	4.13 ± 0.74	0.14	0.0045
10.2 Proper APD operation: understand device structure, principles, functions, programming, and operational procedures	4.60 ± 0.51	0.17	0.0043
10.3 Handling alarms and malfunctions: address common alerts and learn safety precautions during operation	4.40 ± 0.63	0.13	0.0046
10.4 Self-monitoring and documentation: coordinate with remote management systems	4.67 ± 0.82	0.14	0.0047
11. Peritoneal dialysis rehabilitation guidance	4.73 ± 0.59	0.21	0.0047
11.1 Maintain psychological and physical health	4.53 ± 0.64	0.14	0.0049
11.2 Importance of exercise: control body weight, avoid obesity, tailor activities to prevent increased abdominal pressure, ensure secure catheter fixation	4.20 ± 0.86	0.16	0.0044
11.3 Self-management: monitor urine output, ultrafiltration volume, blood pressure, weight, fluid balance; follow medication instructions; replace external catheter every 6 months; attend regular checkups; know the PD emergency hotline	4.47 ± 0.64	0.14	0.0047
11.4 Self-management (duplicate): same content as above for double emphasis in original	4.20 ± 0.68	0.14	0.0689

### Patient validation of content readability

Following internal discussion within the research group, a self-developed “Perioperative Educational Video Feedback Questionnaire for Newly Initiating Peritoneal Dialysis Patients” was disseminated via the Wenjuanxing platform (Appendix 2). After excluding ineligible participants, the questionnaire was administered to 20 patients in the nephrology ward, and all 20 completed it. The questionnaire comprised 20 items, with each rated on a 5-point Likert scale according to perceived importance (1 = not important, 5 = particularly important), yielding a total possible score of 100. Based on team consensus, a score above 80 was considered qualified, and above 90 was rated as excellent. Among the surveys returned, one patient scored in the qualified range, while 19 achieved excellent ratings. Further analysis was performed based on five domains: medication use, home-based care, exit-site and catheter maintenance, dialysis environment and hygiene, and fluid/dietary balance. The mean scores are summarized in Table 2.

**Table 2:** Scores of different dimensions in the patient feedback questionnaire on perioperative educational video.

Dimension	Mean score	Max score	Mi score
Medication	4.90	5	4
Home-based nursing care	4.90	5	4
Exit-site and catheter maintenance	4.90	5	2
Dialysis environment and hygiene	4.95	5	3
Diet and fluid balance	4.61	5	2

### Reliability validation of video content by patient evaluation

#### Theoretical knowledge assessment

Based on the Zhejiang Provincial Peritoneal Dialysis Operational Guidelines, a knowledge assessment questionnaire was independently developed by a PD-specialized nurse and reviewed by the research team (see Appendix 4). Prior to discharge, patients were evaluated using this paper-based test administered by a certified PD-specialized nurse. A score of ≥80 was defined as qualified, and ≥90 as excellent. Assessment results indicated that 4 patients achieved a qualified score and 16 attained an excellent score.

#### Practical skills assessment

The assessment criteria were based on the institutional peritoneal dialysis operation scoring sheet (Appendix 5). The evaluation was conducted by a certified PD-specialized nurse possessing a provincial qualification certificate in peritoneal dialysis issued in Zhejiang Province. Operational competencies were assessed in two areas: exit-site care and peritoneal dialysis fluid exchange techniques. A score ≥85 was considered qualified and ≥90 excellent. Among the 20 patients, 11 were rated qualified, and 9 were rated excellent.

## Discussion

This series of educational videos consists of 11 short videos on different topics. Taking patient acceptability into consideration, the duration of each video is strictly controlled to be under 3 min, and the language used is standardized Mandarin. During the visual design phase, the project team conducted patient interviews and discussions within the peritoneal dialysis (PD) group to determine the animation presentation style. After group members provided optimization suggestions for the design of the animated characters, the final character images were decided. Additionally, to accommodate the cultural differences between patients and their families, the animation content uses simple and easy-to-understand expressions. The narration is done by a professional voice actor in standard Mandarin, with subtitles in regular Microsoft YaHei font, and the colors and font style are carefully coordinated with the visuals to ensure clarity and readability.

To date, no domestic or international research has systematically evaluated the reliability and validity of educational videos in this clinical context, and there is a marked absence of structured perioperative educational content specifically for PD catheterization. This study established a relatively comprehensive and scientifically grounded educational video through literature synthesis, expert consultation, team discussions, and patient interviews. The application of the Analytic Hierarchy Process (AHP) for assigning weight values to video script items further enhanced the methodological robustness of the findings (17). Both rounds of expert consultation achieved a 100% valid response rate, with authority coefficients ≥0.7. After the second round, all Kendall’s coefficients of concordance exceeded 0.3 (*p* < 0.01), indicating high credibility and convergence of expert opinion. Reliability and validity analyses revealed Cronbach’s *α* coefficients >0.9 for all dimensions and the total content, as well as content validity indices (CVI) > 0.9, indicating strong internal consistency across all items. These results confirm that the video possesses a high degree of scientific validity and reliability.

Existing literature, both domestic and international, has addressed PD patient needs related to exit-site care, perioperative nursing, medication adherence, and dietary management. However, the educational video developed in this study offers a more comprehensive approach by addressing the multifaceted needs—physiological, psychological, social, and home-care—of newly initiating PD patients. It provides clinicians and nurses with structured instructional content that enables a more thorough understanding of PD patients’ requirements. Nursing experts affirmed the practical value of wound care and home dialysis procedures depicted in the video, noting their relevance in supporting patient self-management. Psychology experts evaluated the psychological preparation and support components as well-structured and effective in alleviating patient anxiety. Education experts commended the clear structure and accessible language, highlighting the video’s ease of comprehension for patients. Moreover, the expert panel proposed several detailed improvements. For instance, nursing experts recommended enriching the wound care section with additional information on recognizing signs of infection and demonstrating more detailed exchange and exit-site techniques. Psychology experts suggested incorporating more patient communication and disease-related knowledge to ease the psychological burden on patients and families through simplified explanations. Education experts advocated for the integration of interactive components to enhance patient engagement. In response to this feedback, the research team implemented targeted optimizations to ensure the video better addresses the specific needs of newly initiating PD patients.

A multicenter randomized clinical trial in hemodialysis patients found that 50.9% of patients receiving a pain coping skills training video intervention achieved clinically meaningful reduction in pain interference at 12 weeks, significantly outperforming the usual care group (18). Another study in elderly patients with osteoporotic vertebral fractures showed that patients receiving visual health education demonstrated a substantial increase in anti-osteoporosis medication adherence from 28.3 to 76% at 6 months post-surgery (19).

The educational video created in this study aligns with the strategic goals outlined in the national plan by utilizing new media to advance patient education. From the patient’s perspective, video-based instruction is intuitive and accessible, allowing for unlimited, on-demand viewing regardless of time or location. This is particularly advantageous for elderly PD patients, who often have memory and comprehension difficulties; animation-based videos enhance understanding. From the clinical standpoint, the video significantly reduces the time healthcare staff must spend on repetitive instruction, thereby improving efficiency. It may reduce the need for repetitive verbal instructions on basic topics, thereby freeing up healthcare professionals to focus on more complex patient concerns during clinical encounters. Future studies are needed to directly measure the impact of this tool on healthcare workload and time efficiency. Furthermore, the video is likely to enhance the quality and satisfaction of PD care delivery. This study has several limitations. The considerable length of the script may have imposed a cognitive burden on expert reviewers, potentially prompting premature consensus. Additionally, the study was limited to a single institution and a small patient sample, which restricts the generalizability of findings. Future studies with larger sample sizes are necessary to assess the long-term impact of the video on patient disease knowledge, self-care capabilities, and clinical outcomes.

## Conclusion

Through this study, the research team successfully designed a perioperative educational video specifically tailored for newly initiating peritoneal dialysis patients and conducted a comprehensive evaluation of its reliability and validity through multidisciplinary expert consultation. The results demonstrated that the video performed well in terms of content accuracy, practical relevance, and educational effectiveness. It therefore holds substantial application value. Future research directions should include the following: (1) further optimization of video content, particularly through simplification of medical terminology and inclusion of practical skill demonstrations to improve patient comprehension and application; (2) exploration of more interactive instructional modalities, such as the integration of virtual reality to enhance immersive learning experiences; (3) the video offers a promising resource to support patient education in clinical practice. Future research, including randomized controlled trials with baseline and follow-up knowledge assessments, is warranted to evaluate the effectiveness of this video in improving patient knowledge, clinical outcomes, and healthcare utilization; and (4) incorporation of artificial intelligence to create personalized learning paths that deliver customized educational content based on individual patient needs. Further research is warranted to evaluate the impact of the video on quality of life and self-management capabilities among newly initiating peritoneal dialysis patients; (5)We recognize that patients have diverse learning preferences. While the video format primarily caters to visual and auditory learners, we need to integrate it into a broader educational strategy in the future, such as (a) providing a written booklet summarizing key points as a supplement to the video for reading-oriented learners; and (b) having nurses provide hands-on demonstrations and supervised practice for kinesthetic learners during hospitalization.

## Data Availability

The original contributions presented in the study are included in the article/Supplementary material, further inquiries can be directed to the corresponding author.
