# Supplementary material for: Development and validation of an educational video for newly initiating peritoneal dialysis patients: from perioperative care to home-based management
Source: Front Med (Lausanne). 2026 Apr 10;13:1654934. doi: 10.3389/fmed.2026.1654934 (PMC13106050; doi:10.3389/fmed.2026.1654934)
Supplement: Supplementary file 1 [file Table_1.DOCX]

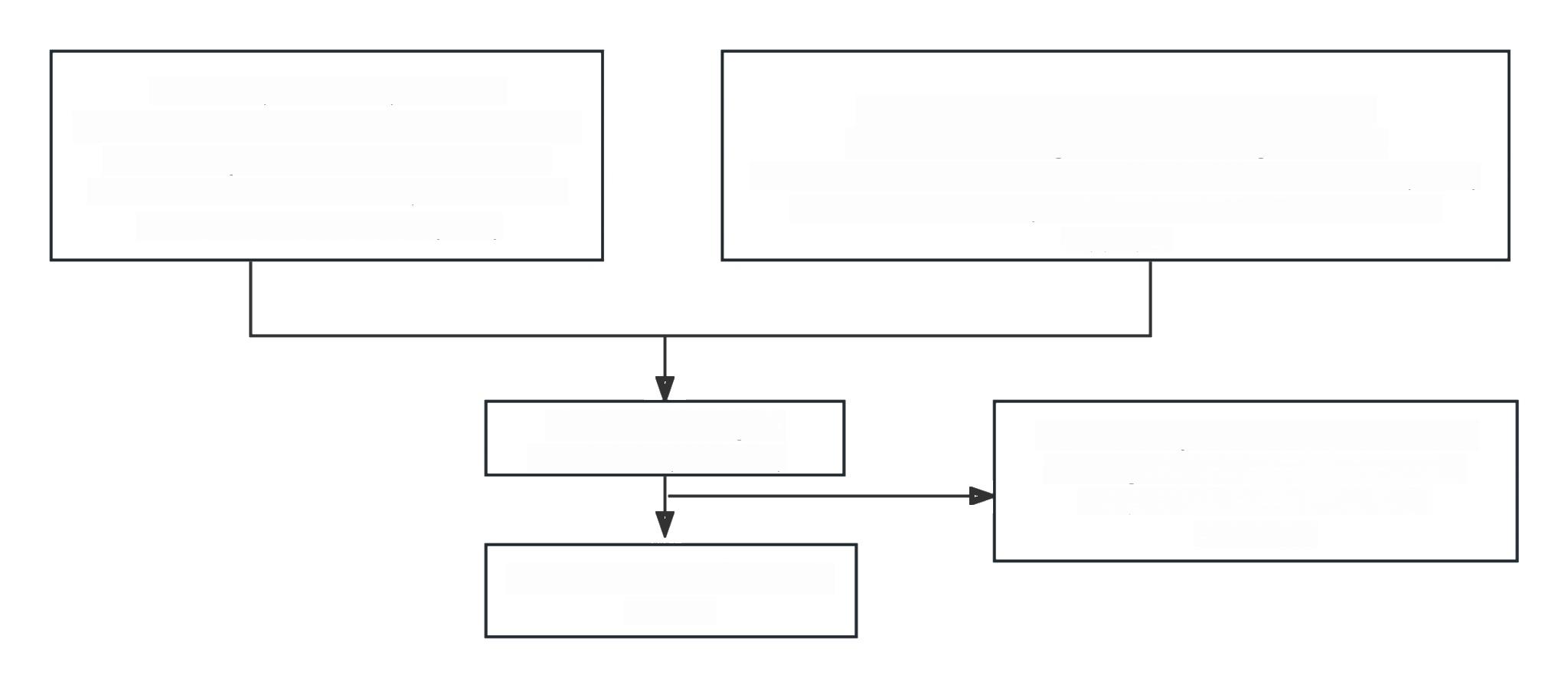
PubMed、CINAHL、Web of

Science、Embase、CochraneLibrary、M

edline、Up To Date、JBI Evidence-

Based Practice Resources、Guidelines

International Network(GIN)

China National Knowledge Infrastructure

(CNKI)、Wanfang Data Knowledge Service

Platform、Chinese Biomedical Literature Databases(CBM)

and other websites/databases、Medlive Guidelines

Network

Initial Screening of Literature(n=1230)

Literature Finally Included

(n=10)

Remove duplicate literature、After

reading the title,abstract and full

text,eliminate the literature

(n=1220)
