# Supplementary material for: Development and validation of an educational video for newly initiating peritoneal dialysis patients: from perioperative care to home-based management
Source: Front Med (Lausanne). 2026 Apr 10;13:1654934. doi: 10.3389/fmed.2026.1654934 (PMC13106050; doi:10.3389/fmed.2026.1654934)
Supplement: Supplementary file 2 [file Table_2.DOCX]

Appendix 2:

**Perioperative Education Video Script for New Peritoneal Dialysis Catheter Patients**

1. Preoperative Knowledge

Xiao Wang is a young man working in the new media industry. He is very busy with his work and often stays up late. Recently, he has been feeling very fatigued, with a poor appetite, nausea and vomiting, and swelling in both of his legs. (He decides to see a doctor.)

Xiao Wang: Doctor, is there something wrong with my kidneys?

Doctor: That is possible. The kidneys continuously filter the blood, removing waste products and excess fluid from the body to maintain health. When kidney function fails, the body’s internal balance is disrupted and warning signals appear. In the early stages of kidney disease, symptoms are not obvious and can be easily overlooked. I recommend that you have some blood and urine tests to help with the diagnosis.

Xiao Wang: Okay.

Doctor: Xiao Wang, your serum creatinine has reached 1200 µmol/L. Combined with your symptoms, this indicates that your kidney disease has progressed to end-stage renal disease.

Xiao Wang: (sad and despairing) What should I do now?

Doctor: Although your kidneys cannot function normally, we can replace their function with hemodialysis, peritoneal dialysis, or kidney transplantation. These therapies are well established in our country. Considering your situation, I recommend peritoneal dialysis, which you can perform at home. Peritoneal dialysis uses your peritoneum as a natural filter: dialysis fluid is introduced into the abdominal cavity and exchanges substances with your blood, supplying what your body needs and removing toxins and excess fluid. There are two main types: CAPD (Continuous Ambulatory Peritoneal Dialysis), which you can do yourself during the day (typically 3–4 exchanges per day, about 30 minutes each); and APD (Automated Peritoneal Dialysis), which uses a machine to perform exchanges automatically, usually at night, making it more suitable for patients who need to work or study during the day.

2. Fluid Balance and Proper Diet

A peritoneal dialysis patient’s body is like a balance scale: having too much or too little fluid upsets the balance, causing discomfort or even threatening life. When there is too much fluid, patients experience weight gain, generalized edema, high blood pressure, and difficulty breathing; when there is too little fluid, they experience weight loss, thirst, low blood pressure, and dizziness. Therefore, controlling fluid balance by accurately calculating daily fluid intake and output is especially important.

Xiao Wang: Are there any methods to control my fluid balance?

Nurse: Follow these four points:

（1）Daily weight: Weigh yourself every morning after emptying your bladder, on an empty stomach. Use the same scale, at the same time of day, and wear similar clothing.

（2）Consistent blood pressure measurement: Measure your blood pressure under fixed conditions each time – the same time of day, the same body location, the same body position, and the same blood pressure device (the so-called “four fixed factors”).

（3）Limit water intake: You can calculate a safe daily fluid amount. Daily required fluid (mL) = yesterday’s urine output + yesterday’s peritoneal dialysate ultrafiltration + 500 mL. (This includes the moisture in all foods, not just drinking water.)

（4）Use higher-concentration dialysate when needed: Use a hypertonic dialysis solution if needed to remove extra water. Since it can be difficult to strictly limit fluid intake, remember these tips: sip water slowly rather than gulping; drink in moderation; use plain water at room temperature; keep a record of your daily intake; avoid carbonated sugary drinks; and don’t forget that fruits and other foods also contain water.

Xiao Wang: They say food is the most important part of life, and following all these rules seems really hard. I’m also concerned about what I can eat.

Nurse: Foods you can eat more freely include: high-quality animal protein (such as yogurt, milk, lean meat, eggs, and fish); foods rich in vitamins; and high-fiber foods (such as whole wheat bread and brown rice). Foods you should limit include: high-phosphorus foods (dairy products, soybeans and related products, animal organs, etc.); high-potassium foods (most fresh vegetables and fruits); and high-sodium foods (salt, MSG, bouillon, and other seasonings). Keep your total daily salt intake below 3 grams (for reference, about 15 mL of soy sauce contains roughly 3 grams of salt). Also limit sweets and fatty foods to avoid weight gain.

3 .Medication Knowledge

Xiao Wang: Doctor, after starting peritoneal dialysis, do I no longer need to take medications?

Doctor: Peritoneal dialysis can replace part of your kidney function, but patients on dialysis can still develop various complications such as hypertension, anemia, and high blood phosphorus. To manage these complications, medication is still necessary. We will create a treatment plan based on your lab results and blood pressure. Most patients need antihypertensive medications, anemia-correcting medications, phosphate binders, and vitamin D supplements.

Xiao Wang: I understand.

Doctor: Both very high and very low blood pressure can be harmful. Patients on peritoneal dialysis who maintain good blood pressure control live significantly longer. Therefore, it is very important to monitor your blood pressure closely while taking antihypertensive medication. Your doctor will adjust your medication as needed – you should not change the dosage on your own. By the way, have you been experiencing any dizziness, fatigue, or shortness of breath with activity lately?

Xiao Wang: Yes. I get very short of breath when climbing stairs.

Doctor: These symptoms are caused by anemia due to reduced erythropoietin production in kidney failure and decreased blood production in the bone marrow. You will need medication to correct the anemia. Common erythropoietin injections should be stored refrigerated at 2–8°C and administered according to the prescription. The oral anemia medication roxadustat is taken three times a week, either on an empty stomach or with food, but it should be spaced at least one hour apart from phosphate binders. Iron supplements should not be taken with tea or coffee.

Xiao Wang: Understood.

Doctor: As peritoneal dialysis continues, high phosphorus levels may occur, causing skin itching and leading to calcification of blood vessels and heart valves, which can result in serious cardiovascular disease and be life-threatening. Therefore, many peritoneal dialysis patients need to take phosphate binders to control blood phosphorus. Common phosphate binders include calcium carbonate, lanthanum carbonate, and sevelamer. Calcium carbonate and lanthanum carbonate should be chewed with meals; sevelamer should be taken with meals as prescribed. Also, do not forget to supplement with vitamin D. In kidney failure, the body cannot produce active vitamin D on its own. Vitamin D acts like a “key” to help calcium enter the bones, keeping them strong.

4. Catheter Car

(Scene: At the doctor’s bedside during rounds; the nurse is performing exit-site care on Xiao Wang, but the adhesive tape is stuck too tightly and won’t come off.)

Xiao Wang: The tape is stuck too tightly. I have scissors. Can I cut it off?

Nurse: That’s a good idea, but you must not use scissors!

Xiao Wang: Why not?

Doctor: Xiao Wang, what you’re doing is incorrect. A dialysis catheter is a lifeline for peritoneal dialysis patients. It is the channel through which dialysis fluid enters and exits the peritoneal cavity. (Picture of the catheter.) You cannot replace it. Using a sharp object could accidentally cut or puncture the catheter.

Xiao Wang: Ah! That’s so serious?

Doctor: Yes! Protecting the dialysis catheter is extremely important. Cutting or puncturing it can lead to peritonitis or require catheter replacement. If that happens, you must immediately contact a specialist doctor or nurse for professional guidance.

Xiao Wang: Thank you for the reminder. I will remember.

Nurse: Very good. In addition, before touching the catheter, you must wash your hands. Do not use alcohol to wipe the catheter, as that can cause aging. The catheter must always be fixed to the skin, and not be pulled, folded, or twisted.

5. Exit-Site Care

(Scene: It’s hot. Xiao Wang is sweating all over. On postoperative day 4, he takes a shower without any protective measures and just quickly disinfects and changes the dressing. On postoperative day 6, he feels pain at the exit site and seeks the doctor’s help.)

Xiao Wang: Doctor, my exit site is very painful.

Doctor: (The doctor begins examining the exit site.) Your exit site has purulent discharge, the skin is red, and it’s painful on pressure. These signs are consistent with exit-site infection. Did you take a shower or change the dressing properly recently?

Xiao Wang: Uh! (Embarrassed) I just took a shower a couple of days ago and simply changed the dressing.

Doctor: Your exit-site infection is closely related to that shower. Within 2 weeks postoperatively, you cannot take a shower (only sponge baths). Between 2–6 weeks, you can shower under the protection of a sterile dressing.
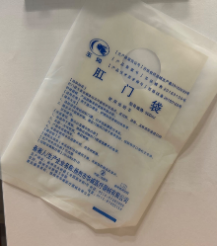
After 6 weeks, if well healed, you can shower without protection. Do not take tub baths. After each shower, immediately perform exit-site care.

Xiao Wang: Mm, I understand. What should I do next?

Doctor: Proper exit-site dressing changes are very important. Before changing the dressing, wash your hands and put on a mask. Prepare your supplies. First, remove the old dressing and examine the exit site for redness or swelling. Next, press along the tunnel direction to check for discharge. Then wash your hands again. Disinfect from the center outward in a circular motion three times, covering a larger area than the dressing. Wait for it to dry, cover with a new dressing, and fix the extension set securely.


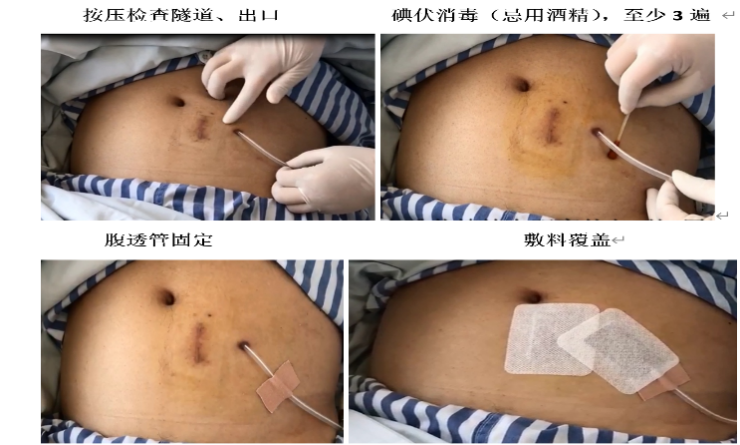


6 .Peritoneal Dialysis Fluid Exchange Procedure

(Scene: The patient underwent peritoneal dialysis surgery yesterday and is lying in bed. The nurse is standing at the bedside.)

Nurse: Xiao Wang, you just had surgery yesterday. How do you feel today? Does the wound hurt?

Xiao Wang: I feel fine, but the wound hurts a little.

Nurse: Mild pain at the surgical site is normal. No need to worry. Your wound is not bleeding. We recommend getting out of bed and moving around.

Xiao Wang: Okay.

Nurse: Peritoneal dialysis is done at home, and fluid exchange is very important. You must master the procedure!

Xiao Wang: Sigh! I’m afraid I won’t learn it. (Worried expression)

Nurse: Don’t worry. The process is quite simple and mainly includes six steps:

(1).Preparation: Close doors and windows, turn off fans and air conditioners, wipe the table, wash your hands using the seven-step handwashing technique (picture of handwashing), put on a mask, prepare the blue clamp, the disinfected cap, and the dialysis fluid. Check the dialysis fluid’s expiration date, concentration, temperature, and clarity.

(2.)Connection: Hang the dialysis fluid on the hook and connect the extension set to the fluid bag’s tubing.

(3).Drainage: Open the clamp on the extension set to start draining. When drainage is complete, close the clamp.

(4).Rinse: Clamp the outflow tubing with the blue clamp. Confirm the extension set clamp is closed, break off the green break-off point, open the blue clamp to expel air, then close the blue clamp.

(5).Infusion: Open the extension set clamp and infuse the dialysis fluid into the peritoneal cavity. When infusion is complete, close the clamp.

(6) .Disconnection: Confirm that the inflow tubing, outflow tubing, and extension set clamp are all closed. Check the disinfected cap’s expiration date and packaging integrity, then disconnect the tubing and place the disinfected cap on the extension set’s connector. Check the dialysis fluid’s color and appearance. Weigh and record the ultrafiltration volume.

Companion (Mother): I remember a bit after watching once. I will supervise Xiao Wang to practice more.

Nurse: Practice makes perfect. Doing it several times will help. In addition, prepare a clean, dry room with good lighting, do not keep pets, disinfect the floor and table with alcohol or chlorine-based disinfectant twice a day, disinfect with ultraviolet light twice a day for 30 minutes each time, then open windows for ventilation for 5 minutes. Once a week, use anhydrous alcohol to wipe the light fixtures, and replace light bulbs after 1000 hours. Do not answer phone calls during the procedure. Dialysis fluid temperature should be 37°C. When warming, do not open the outer bag. Only dry warming; do not warm in water.

7. Simulated Home Dialysis Issues – Abnormal Fluid Exchange

Home peritoneal dialysis may encounter various issues. Learning how to handle them will solve these problems easily.

Xiao Wang: Doctor, a few days ago when I was performing dialysis, the fluid wouldn’t flow in. I was very anxious and called for the nurse. It turned out I hadn’t opened the clamp. Haha, it was a bit embarrassing.

Doctor: When fluid infusion or drainage is difficult, check whether the dialysis fluid bag is higher than your abdomen, whether the drainage bag is lower than your abdomen, whether the tubing is kinked, whether the clamp is open, and whether there are air bubbles in the tubing. If there are air bubbles, flick them upward with your finger. If there is fibrin in the tubing, gently squeeze the bag or wrap the tubing around your finger three times and squeeze firmly. If these methods are ineffective, contact your doctor or nurse.

Nurse: If none of the doctor’s suggestions work, you can try changing your position—sit, stand, lie on your left or right side—and see if drainage improves.

Xiao Wang: Oh, I see. There are so many methods.

Doctor: If you have habitual constipation, you also need to take laxatives to avoid catheter migration.

Xiao Wang: Okay. I have another concern: I’ve just started dialysis and am not skilled yet. What if I touch a sterile area during the procedure?

Doctor: The sterile areas are the extension set connector, the dual-flow fluid connector, and the inside of the disinfected cap. If you touch the dual-flow fluid connector or the inside of the disinfected cap, discard them and replace them with new ones.
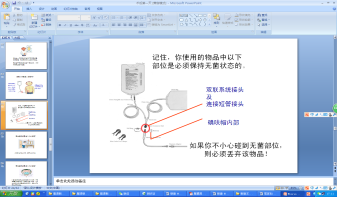


Companion (Mother): What if the extension set connector is contaminated?

Doctor: If the extension set connector is contaminated, cover it with the disinfected cap and immediately go to the nearest dialysis center to replace the extension set.

Xiao Wang: A few days ago, a dialysis patient friend mentioned fluid leakage during dialysis, and I only heard a little about it.

Doctor: Causes of leakage include rupture of the dual-flow fluid tubing, poor closure at the connection of the extension set, rupture of the peritoneal catheter, and leakage at the exit site. Nurses have more experience with these.

Nurse: If the dual-flow fluid tubing ruptures, immediately close the extension set clamp. Use two blue clamps to clamp both ends of the rupture. Replace with a new bag of dialysis fluid and continue dialysis. If the extension set connection is not closed properly, clamp the proximal end of the extension set with a blue clamp and return to the dialysis center to replace it. If the peritoneal catheter ruptures, immediately clamp the catheter near the rupture with a blue clamp, return to the dialysis center for disinfection and treatment, and replace the catheter if necessary. If leakage at the exit site occurs, drain any fluid from the peritoneal cavity, cover the exit site with sterile gauze, and immediately return to the dialysis center for treatment.

Xiao Wang & Companion (Mother) (in unison): There’s so much to learn; we need to absorb it slowly.

Doctor: During fluid exchange, the extension set or titanium connector may come off. Immediately clamp the peritoneal catheter near the peritoneum with a blue clamp and return to the dialysis center for treatment.

8.Simulated Home Dialysis Issues – Living

Nurse: Xiao Wang, do you know what dangers constipation can pose to peritoneal dialysis patients?

Xiao Wang: Constipation can cause dangers as well?

Nurse: Of course. Constipation can cause the colon to dilate due to fecal impaction, and the dilated colon can compress the peritoneal catheter, causing infusion and drainage problems. During constipation, bacteria may also enter the peritoneal cavity, causing peritonitis.

Xiao Wang: I never imagined that simple constipation could cause such big dangers. So, can I eat more high-fiber foods, like whole wheat bread and high-fiber cereal, and exercise daily to prevent constipation?

Nurse: Absolutely. If there is constipation, you can also ask the doctor to prescribe mild laxatives to relieve constipation.
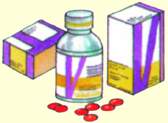


Xiao Wang: Prevent problems before they happen. I will take it seriously. Today I met a dialysis patient who has been on peritoneal dialysis for 5 years. He said he suffered from severe skin itching, scratching until he had wounds all over, which looked a bit scary.

Nurse: We learned about skin itching caused by hyperphosphatemia. That is his reason.

Xiao Wang: Is there a way to relieve the itching?

Nurse: You can eat fewer high-phosphorus foods and take phosphate binders as prescribed to lower serum phosphorus. Do not use strongly scented soaps or harsh cleansers. After showering, use some moisturizer, but do not apply it at the catheter exit site.

Xiao Wang: Then I need to control what I eat and eat fewer high-phosphorus foods.

Nurse: Very good. Have you heard about hernias?

Xiao Wang: Yes.

Nurse: Peritoneal dialysis patients can develop hernias too. Most occur when strenuous activity or lifting heavy objects causes increased intra-abdominal pressure. During peritoneal dialysis, a large amount of dialysis fluid enters the abdominal cavity, increasing intra-abdominal pressure and making hernias more likely. They can be “repaired” by simple surgery. If you suspect you have a hernia, contact your dialysis center.

9.Simulated Home Dialysis Issues – Infection

Infection is everywhere: surgical site infections, post-COVID lung infections... Peritoneal dialysis’s most common infections are peritonitis and exit-site infections.


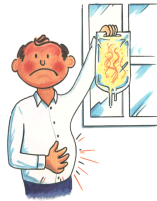


Doctor: Do you know what the symptoms of peritonitis are and how to recognize them?

Xiao Wang: Abdominal pain, fever... I’m not particularly clear on the others.

Companion (Mother): Cloudy dialysis fluid.

Doctor: All correct. Peritonitis includes five symptoms: cloudy dialysate, abdominal pain, fever, nausea and vomiting, and diarrhea. Sometimes one or more of these appear. You need to know all the symptoms to detect peritonitis promptly.

Companion (Mother): If peritonitis occurs, what should I do?

Doctor: Immediately contact your hospital and clamp the first bag of cloudy dialysate with a blue clamp. Bring the entire bag of dialysate (do not use scissors to cut a small sample or take only part of it) to the hospital for testing. The hospital will take a portion of the cloudy dialysate for testing to diagnose peritonitis and provide timely empirical treatment.


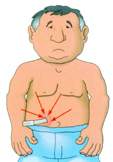


Xiao Wang: Mm, I will operate correctly to avoid peritonitis. I learned about exit-site infection symptoms from the previous lessons. If the exit site is infected, do I also need to contact you first?

Doctor: Yes. If you notice any one of the four symptoms, you should immediately call the dialysis center. Bacteria at the exit site can travel along the catheter into the peritoneal cavity, causing peritonitis. If pressing the subcutaneous tunnel area causes pain, it could indicate a tunnel infection, which also requires prompt reporting and treatment, otherwise it can more easily lead to peritonitis.

Nurse: Proper exit-site care can prevent infections. Always remember to wash your hands before touching the catheter.

10 .APD (Automated Peritoneal Dialysis Cycler) Operation

Scene: In the ward, Xiao Wang consults the nurse.

Xiao Wang: Nurse, besides manual peritoneal dialysis, is there a machine-based way?

Nurse: Yes, we call it an Automated Peritoneal Dialysis Cycler (machine picture). This is a method that uses a machine to automatically control dialysate inflow and outflow into the peritoneal cavity. It can be done at night and is simple and convenient to operate.

Xiao Wang: Would that suit me?

Nurse: It certainly would. The cycler is mainly for patients who have study or work needs and cannot perform multiple exchanges during the day, newly catheterized patients or those who need more frequent dialysis, anuric patients, patients with large body size, pediatric patients, and elderly patients who need assistance.

Xiao Wang: Is machine operation the same as manual operation?

Nurse: There are some differences.

(Treatment start) Prepare necessary items: dialysis fluid, disinfected cap, waste bag, and the cycler tubing set; wash hands, put on a mask; plug in and turn on the machine—set the prescription (doctor can set or change remotely)—check the dialysis fluid and place it on the warming plate—install the cycler tubing, and the machine performs self-check—connect the dialysis fluid and venting—connect the patient’s tubing, and start therapy.

(Treatment end) When the interface shows “treatment completed,” close all clamps and disconnect tubing. Cover the extension set with the disinfected cap, remove the cycler tubing—turn off the machine, unplug it—then check the dialysis fluid’s color and appearance, record the ultrafiltration volume, pour the drained fluid into the toilet, rinse and dry the waste bag, discard the dialysate bags and cycler tubing, and wipe and disinfect the cycler with disinfectant wipes.

11.Peritoneal Dialysis Rehabilitation Guidance

Nurse: Xiao Wang, congratulations on passing the theoretical and practical exams for peritoneal dialysis. You will be discharged soon!

Xiao Wang (Happy): Your patient guidance taught me so much knowledge.

Nurse: After discharge, continue to maintain a positive and optimistic attitude. If your body allows, you can continue to work or study, but balance work and rest. Stay confident and do not let the disease bring you too much stress. Share and communicate with family and friends to relieve your stress.

Xiao Wang: Don’t worry! I will plan my work and life well so I won’t be as exhausted as before.

Nurse: You used to be busy with work and lacked exercise. Actually, exercise is very important for peritoneal dialysis patients. It has many benefits: it helps improve nutritional status, maintain strong bones, control weight, stabilize blood pressure and blood sugar, improve mental state, and enhance immunity.

Xiao Wang: What kind of exercise can we dialysis patients do?

Nurse: You can take half an hour to an hour each day to exercise. The type of exercise varies by person. Generally, choose low-to-moderate intensity activities, such as walking, practicing Tai Chi, playing table tennis, etc. Choose according to your preference. During exercise, avoid increasing abdominal pressure and keep the extension set fixed.

Nurse: Xiao Wang, self-management is something every patient must do but finds difficult: maintain fluid intake and output balance every day, record urine volume, peritoneal dialysis ultrafiltration volume, weight, and blood pressure in the dialysis record book. Also maintain standard fluid exchange and dressing change procedures, take medications as prescribed, and detect and treat dialysis complications early. Finally, you need regular outpatient follow-up, recheck complete blood count, blood chemistry, 24-hour urine chemistry, and peritoneal dialysis fluid chemistry; replace the peritoneal dialysis external extension set every 6 months.

Xiao Wang: Maintaining fluid intake and output balance every day is indeed very difficult, but to reduce hospital visits and minimize the suffering caused by complications, I am willing to work hard.

Nurse: Xiao Wang, you will certainly become a persistent and determined dialysis patient. Keep it up!
