# Supplementary material for: Development and validation of an educational video for newly initiating peritoneal dialysis patients: from perioperative care to home-based management
Source: Front Med (Lausanne). 2026 Apr 10;13:1654934. doi: 10.3389/fmed.2026.1654934 (PMC13106050; doi:10.3389/fmed.2026.1654934)
Supplement: Supplementary file 3 [file Table_3.DOCX]

Appendix 3:

**Feedback Questionnaire for Perioperative Education Videos for New Peritoneal Dialysis Catheter Patients**

Dear Patient, through our video lessons, we hope you have learned about peritoneal dialysis procedures and related precautions. Please take a few minutes to complete the following questionnaire. Your feedback and suggestions are valuable to us and will help us continue to improve our service. Please rate the importance of each topic below on a scale from 1 to 5 (1 = Not important at all, 2 = Not important, 3 = Somewhat important, 4 = Important, 5 = Very important).

1.Do you think the educational content presented in the 11 videos is important to you overall?

(1) Not important at all (2) Not important (3) Somewhat important (4) Important (5) Very important

2.After watching the videos, do you consider the environment used for peritoneal dialysis exchanges to be important for you?

(1) Not important at all (2) Not important (3) Somewhat important (4) Important (5) Very important

3.After watching the videos, do you think the location where you perform fluid exchange is important for preventing peritonitis?

(1) Not important at all (2) Not important (3) Somewhat important (4) Important (5) Very important

4.After watching the videos, do you think it is important to wash your hands and wear a mask before each exchange procedure?

(1) Not important at all (2) Not important (3) Somewhat important (4) Important (5) Very important

5.Do you think changing your exit-site dressing on schedule is important?

(1) Not important at all (2) Not important (3) Somewhat important (4) Important (5) Very important

6.After watching the videos, do you think it is important to secure the peritoneal catheter with a protective belt at all times?

(1) Not important at all (2) Not important (3) Somewhat important (4) Important (5) Very important

7.Do you think strictly following intake guidelines to control your daily water intake is important?

(1) Not important at all (2) Not important (3) Somewhat important (4) Important (5) Very important

8.After watching the videos, will you strictly control your water intake as required?

(1) Not important at all (2) Not important (3) Somewhat important (4) Important (5) Very important

9.After watching the videos, do you think learning how to handle infusion difficulties or poor drainage is important?

(1) Not important at all (2) Not important (3) Somewhat important (4) Important (5) Very important

10.Do you think maintaining sterility of items and procedures during the operation is important?

(1) Not important at all (2) Not important (3) Somewhat important (4) Important (5) Very important

11.Do you think recognizing the early symptoms of peritonitis is important?

(1) Not important at all (2) Not important (3) Somewhat important (4) Important (5) Very important

12.Do you think it is important to promptly visit the hospital upon noticing early symptoms of peritonitis?

(1) Not important at all (2) Not important (3) Somewhat important (4) Important (5) Very important

13.Do you think understanding the signs of exit-site infection is important?

(1) Not important at all (2) Not important (3) Somewhat important (4) Important (5) Very important

14.Do you think keeping bowels regular and preventing constipation is important for dialysis patients?

(1) Not important at all (2) Not important (3) Somewhat important (4) Important (5) Very important

15.Do you think replacing the peritoneal dialysis extension set on schedule is important?

(1) Not important at all (2) Not important (3) Somewhat important (4) Important (5) Very important

16.Do you think coming to the hospital for regular follow-up is important?

(1) Not important at all (2) Not important (3) Somewhat important (4) Important (5) Very important

17.Do you think carefully filling out and keeping your peritoneal dialysis diary is important?

(1) Not important at all (2) Not important (3) Somewhat important (4) Important (5) Very important

18.Do you think accurately recording your daily urine volume is important?

(1) Not important at all (2) Not important (3) Somewhat important (4) Important (5) Very important

19.Do you think proper diet control is important for peritoneal dialysis patients?

(1) Not important at all (2) Not important (3) Somewhat important (4) Important (5) Very important

20.Do you think taking your medications on time and as prescribed is important for controlling your disease?

(1) Not important at all (2) Not important (3) Somewhat important (4) Important (5) Very important
