# Supplementary material for: Development and validation of an educational video for newly initiating peritoneal dialysis patients: from perioperative care to home-based management
Source: Front Med (Lausanne). 2026 Apr 10;13:1654934. doi: 10.3389/fmed.2026.1654934 (PMC13106050; doi:10.3389/fmed.2026.1654934)
Supplement: Supplementary file 4 [file Table_4.DOCX]

Appendix 4:

| The Second Affiliated Hospital, Zhejiang University School of Medicine  **Assessment Questionnaire for Peritoneal Dialysis Center**  Name: __________　Medical Record No.: __________　Score: ____　Date: __________  **I. Multiple Choice Questions (5 points each)**  1.Which items absolutely must not be touched during the procedure? | | | |
| --- | --- | --- | --- |
| 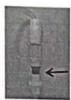  A | 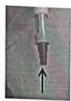  B | 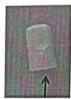  C | 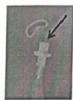  D |
| 2.Which of the following can be symptoms of peritonitis?  A: Nausea and vomiting  B: Diarrhea  C: Decreased ultrafiltration volume  D: Cloudy dialysate  E: Fever  F: Abdominal pain  3.If peritonitis is suspected, what should you do?  A: Continue dialysis for two more days and wait  B: Immediately call the dialysis center  C: Collect a small sample of dialysate and send it to the center for testing  D: Bring the entire dialysate bag to the center for testing  4.Which items must be monitored every day?  A: Daily weight measurement  B: Blood pressure measurement at a fixed time each day  C: Daily calculation of ultrafiltration and urine volume  D: Daily assessment of fluid (intake) volume  5.If your weight changes by how many kilograms in a short time, you must contact your responsible nurse?  A: 1 kg B: 2 kg C: 5 kg D: No need to contact  6.If the dialysate will not infuse, what do you do?  A: Check if the cap is opened  B: Check if the roller clamp on the extension set is open  C: Check if the catheter tubing is kinked or twisted  D: Check for any air or fibrin clots blocking the line  7.If drainage is difficult, what do you do?  A: Check if the spiral clamp on the extension set is open  B: Check for any air or fibrin clots blocking the line  C: Check if the drainage bag is placed lower than the abdomen  D: Check if your body position is correct  E: Check for constipation or intestinal gas  8.If ultrafiltration suddenly decreases, which should be considered?  A: Tubing blockage  B: Kinking or twisting of the catheter  C: Peritonitis  D: Constipation  E: Catheter displacement  9.What is the recommended daily salt limit for a dialysis patient?  A: 1 g B: 2 g C: 3 g D: 4 g E: 5 g  10.How should phosphate binders be taken?  A: Take before meals  B: Take after meals  C: Take during meals (swallowed)  D: Chew during meals  11. What are the symptoms of an exit-site infection?  A: Redness of the skin at the exit site  B: Swelling at the exit site  C: Pain at the exit site  D: Purulent (pus-like) discharge at the exit site  E: Fever  12. What items should you bring to your follow-up visit?  A: Dialysis record book  B: Three-day dietary record  C: Monthly outpatient questionnaire  D: Blood sugar record book  **II. True/False Questions (5 points each)**  13. After catheter placement, you cannot move or work.  True / False   1. Because you worry the exposed connector gets dirty, you wipe it with povidone-iodine every day.   True / False  15. Six months after the operation, you can shower with water directed at the exit site.  True / False  16.After taking a shower, you still need to perform exit-site care.  True / False  17. Whether for outpatient follow-up, medication refill, or any outpatient issue, you need to register again.  True / False  **III. Short Answer Questions (15 points each)**  18. If the clamp is closed and the end of the extension set (titanium connector) touches other objects, what should you do?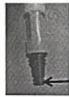  19.Do you know the name of your peritoneal dialysis nurse? Please fill in:  20.Are you satisfied with the training method used in the ward?  A .Very satisfied　B. Mostly satisfied　C. Not satisfied  21Do you have any suggestions or comments about our training methods and content? | | | |
