# Supplementary material for: Development and validation of an educational video for newly initiating peritoneal dialysis patients: from perioperative care to home-based management
Source: Front Med (Lausanne). 2026 Apr 10;13:1654934. doi: 10.3389/fmed.2026.1654934 (PMC13106050; doi:10.3389/fmed.2026.1654934)
Supplement: Supplementary file 5 [file Table_5.DOCX]

Appendix 5:

**Peritoneal Dialysis Procedure Competency Assessment (Revised February 2022)**

Name: __________　Medical Record No.: __________　Score: ____　Date: __________

|  | Project | Operation Problem | Deduct Points |
| --- | --- | --- | --- |
| Before O peration | Environment Preparation (Verbal) | ※Did not perform ultraviolet (UV) disinfection at home | 5 |
|  |  | UV disinfection duration less than 30 minutes | 2 |
|  |  | Less than 2 UV disinfections per day | 2 |
|  |  | Did not use 70%-alcohol to wipe light fixtures weekly | 2 |
|  |  | Did not open windows/doors for ventilation for 5 minutes after UV disinfection | 2 |
|  |  | Did not keep pets away from the procedure area | 2 |
|  |  | Procedure area not located away from fans, windows, or air conditioners | 2 |
|  |  | Answered phone calls or opened doors during the procedure | 2 |
|  | Personnel Preparation | Clothing not clean or tidy | 2 |

|  |  | Fingernails dirty or longer than 2 mm | 2 |
| --- | --- | --- | --- |
|  |  | ※Did not use soap (or hand sanitizer) to wash hands | 5 |
|  |  | Did not use the seven-step handwashing technique | 2 |
|  |  | Total handwashing time less than 2 minutes | 2 |
|  |  | ※（Patient or caregiver ）did not wear a mask at home | 5 |
|  |  | Mask worn incorrectly | 2 |
|  | Supplies Preparation | Supplies not fully prepared before procedure | 2 |
|  |  | Dialysis fluid not warmed properly | 2 |
| During Operation | Single-use Items Inspection | Did not check dialysis solution (expiry date, concentration, clarity, leaks, temperature) | 5 |
|  |  | Did not check disinfectant cap (expiry date, integrity) | 2 |
|  | Connection Technique | ※Contaminated the sterile connection when connecting | 10 |
|  |  | Sterile connector not held upright during connection | 2 |
|  |  | Poor connection technique with potential contamination | 5 |
|  |  | Did not remove air from the dialysis tubing | 5 |
|  |  | ※Contaminated the catheter while placing the disinfectant cap | 5 |
|  | Overall Proficiency | Overall procedure performance was not proficient | 6 |
| After Operation | Tube fixation | Extension set not secured properly after dialysis | 2 |
|  | Check the dialysis fluid | ※Did not check the color and appearance of the drained fluid | 10 |
|  | Calculate the ultrafiltration volume | Did not correctly calculate ultrafiltration volume | 5 |
| Score | | |  |

Notes：Items marked with “※” result in immediate failure and require re-assessment. A total score below 85 requires re-examination.
